# HIV-1 Nef Binds the DOCK2–ELMO1 Complex to Activate Rac and Inhibit Lymphocyte Chemotaxis

**DOI:** 10.1371/journal.pbio.0020006

**Published:** 2004-01-20

**Authors:** Ajit Janardhan, Tomek Swigut, Brian Hill, Michael P Myers, Jacek Skowronski

**Affiliations:** **1**Cold Spring Harbor Laboratory, Cold Spring HarborNew YorkUnited States of America; **2**Program in Genetics and Medical Scientist Training Program, Stony Brook UniversityStony Brook, New YorkUnited States of America

## Abstract

The infectious cycle of primate lentiviruses is intimately linked to interactions between cells of the immune system. Nef, a potent virulence factor, alters cellular environments to increase lentiviral replication in the host, yet the mechanisms underlying these effects have remained elusive. Since Nef likely functions as an adaptor protein, we exploited a proteomic approach to directly identify molecules that Nef targets to subvert the signaling machinery in T cells. We purified to near homogeneity a major Nef-associated protein complex from T cells and identified by mass spectroscopy its subunits as DOCK2–ELMO1, a key activator of Rac in antigen- and chemokine-initiated signaling pathways, and Rac. We show that Nef activates Rac in T cell lines and in primary T cells following infection with HIV-1 in the absence of antigenic stimuli. Nef activates Rac by binding the DOCK2–ELMO1 complex, and this interaction is linked to the abilities of Nef to inhibit chemotaxis and promote T cell activation. Our data indicate that Nef targets a critical switch that regulates Rac GTPases downstream of chemokine- and antigen-initiated signaling pathways. This interaction enables Nef to influence multiple aspects of T cell function and thus provides an important mechanism by which Nef impacts pathogenesis by primate lentiviruses.

## Abstract 2

Upon HIV-1 infection of T cells, Nef activates Rac by binding the DOCK2-ELMO1 complex. In this way, Nef influences multiple aspects of T cell function, including inhibition of lymphocyte chemotaxis

## Introduction

Primate lentiviruses persist in the host by active replication and can reemerge from latent reservoirs that are established in cells of the immune system (Finzi and Siliciano 1998; Douek et al. 2003). The infectious cycle is intimately linked to interactions between circulating T cells and antigen-presenting cells (Stevenson et al. 1990; Bukrinsky et al. 1991; Embretson et al. 1993; Swingler et al. 1999; Geijtenbeek et al. 2000). These interactions involve T cell migration, adhesion, and antigen-initiated signaling, processes that are dependent on cytoskeletal dynamics regulated by the Rho subfamily of small GTPases (Hall 1998; Schmitz et al. 2000).

The lentiviral accessory protein Nef is a multifunctional regulator that is important for rapid progression to AIDS (Kestler et al. 1991; Piguet et al. 1999; Renkema and Saksela 2000). One key function of Nef is its ability to facilitate activation of infected cells and thus provide an environment that is conducive for viral replication (Skowronski et al. 1993; Baur et al. 1994; Du et al. 1996; Schrager and Marsh 1999; Simmons et al. 2001). Another important function of Nef is its ability to promote evasion of the antiviral immune response. This is accomplished by downregulation of class I MHC complexes from the surface of infected cells, which protects against detection by cytoxic T cells specific for viral antigens (Schwartz et al. 1996; Collins et al. 1998).

The ability of Nef to facilitate T cell activation is well documented. Thymic and peripheral CD4^+^ T cells from transgenic mice are hypersensitive to stimulation via the T cell antigen receptor (TCR) (Skowronski et al. 1993; Hanna et al. 1998), as are resting primary human CD4^+^ T cells (Schrager and Marsh 1999; Wang et al. 2000) and cell lines transduced to express HIV-1 Nef (Alexander et al. 1994; Baur et al. 1994). Nef was reported to associate with molecules that play important roles in antigen-initiated signaling in T cells, including elements of signaling pathways involving small GTPases. Specifically, Nef was reported to associate with Vav (Fackler et al. 1999) and activate p21-activated serine–threonine kinases (PAKs), possibly though the activation of Rac or CDC42 (Lu et al. 1996). Recent observations that Nef can activate Rac in a glial cell line have strengthened the connection between Nef and these pathways (Vilhardt et al. 2002). The notion that effects of Nef on signaling machineries in T cells are mediated by small GTPases, their effectors, or both represents an attractive possibility, yet the exact mechanism resulting in activation of these pathways has remained elusive. Since Nef likely functions as an adaptor protein, we exploited a proteomic approach to directly identify the key molecules Nef uses to subvert the signaling machinery in T cells. Here we show that Nef targets a key activator of Rac GTPases that functions downstream of the TCR and chemokine receptors.

## Results

### Nef Binds DOCK2, ELMO1, and Rac in T Cells

**Figure 1:**
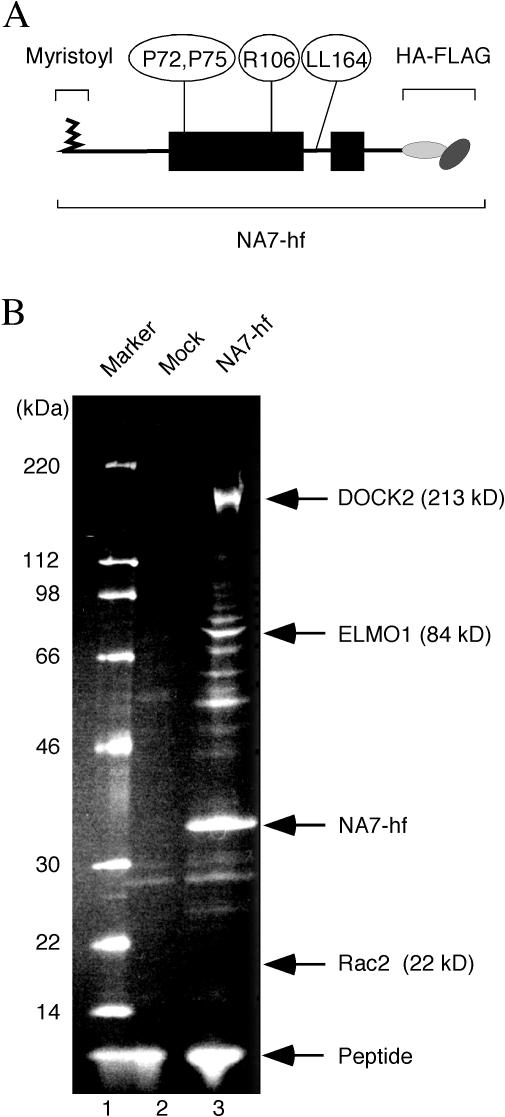
DOCK2, ELMO1, and Rac Are Abundant Nef-Associated Proteins in T Cells (A) Schematic representation of epitope-tagged HIV-1 Nef (NA7-hf). The structured regions of Nef are boxed and the disordered regions, as determined by X-ray crystallography and NMR studies, are shown by a thin line. The locations of the N-terminal myristoyl moiety, prolines P72 and P75 in the PP-II helix, arginine R106, leucines L164 and L165 (LL164), and the C-terminal HA-FLAG epitopes are indicated. (B) DOCK2, ELMO1, and Rac2 specifically copurify with HIV-1 Nef from Jurkat T cells. Jurkat T cells (1.8 ×10^10^) stably expressing NA7-hf (lane 3) or control Jurkat cells (lane 2) were subjected to the two-step immunopurification procedure described in the text (see Materials and Methods). Polypeptides present in purified immune complexes were resolved by SDS-PAGE and analyzed by LC/MS/MS. We identified 58 DOCK2-specific peptides covering 869 out of 1830 total amino acid residues (47.5% coverage, expectation value 6.0 × 10^–130^), 10 ELMO1-specific peptides covering 122 out of 727 total amino acid residues (16.8% coverage, expectation value 1.0 × 10^−10^), and three Rac-specific (two of which were Rac2-specific) peptides covering 26 out of 192 total amino acid residues (13.5% coverage, expectation value 4.6 × 10^−4^). Bands corresponding to DOCK2, ELMO1, Rac2 and their predicted molecular weights, NA7-hf Nef, and the FLAG peptide used for elution are indicated.

To identify downstream effectors of Nef in T lymphocytes, we generated CD4^+^ Jurkat T cells that stably express the extensively studied patient-derived HIV-1 Nef protein NA7 (Mariani and Skowronski 1993) tagged at its C-terminus with HA and FLAG epitopes (NA7-hf) (Figure 1A). Nef and its associated proteins were purified by successive immunoprecipitations with anti-HA- and then anti-FLAG epitope antibodies, followed each time by elution with the respective peptide epitope and resolved by SDS-PAGE. Several polypeptides with apparent molecular weights ranging from approximately 20 kDa to 220 kDa copurified with HIV-1 Nef, but were absent in preparations from control cells that do not express Nef (Figure 1B). Gel slices containing these polypeptides were digested with trypsin, and the resulting peptides were sequenced by liquid chromatography tandem mass spectroscopy (LC/MS/MS) and subjected to database searches (Hu et al. 2002). Two abundant Nef-associated proteins, DOCK2 and ELMO1, were thus identified. DOCK2 is a lymphocyte-specific CED5/DOCK180/Myoblast City (CDM) family protein that regulates the activity of Rac1 and Rac2 GTPases downstream of chemokine receptors and the TCR and is essential for lymphocyte migration and normal antigen-specific responses of T cells (Fukui et al. 2001; Reif and Cyster 2002; Sanui et al. 2003a). Rac GTPases are members of the Rho subfamily of small GTP-binding proteins that control several processes, including cytoskeletal rearrangements during cell motility and T cell activation (Hall 1998). Recent studies showed that ELMO1 functionally cooperates with CDM family proteins to activate Rac (Brugnera et al. 2002; Sanui et al. 2003b). Significantly, our mass spectroscopic analyses of Nef-associated proteins also identified Rac2. Furthermore, in addition to Rac2-specific peptides, we also detected peptides shared by Rac1 and Rac2, raising the possibility that Rac1 also associates with HIV-1 Nef in T cells. The ubiquitously expressed Rac1 and hematopoietic cell-specific Rac2 are 95% identical, and both isoforms regulate cytoskeletal dynamics and gene expression in T lymphocytes (Yu et al. 2001; Croker et al. 2002).

### Nef Binds the DOCK2–ELMO1–Rac Complex

**Figure 2:**
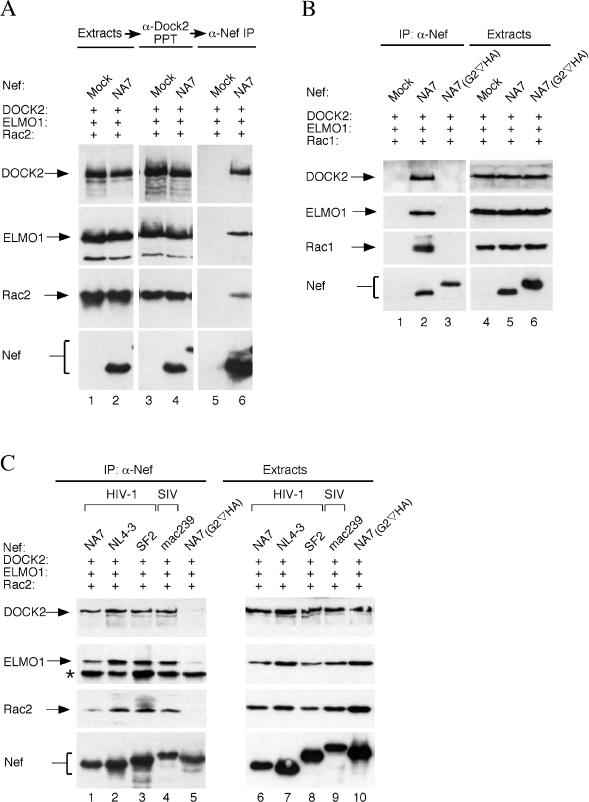
Lentiviral Nef Binds the DOCK2–ELMO1–Rac Complex (A) HIV-1 Nef binds the DOCK2–ELMO1–Rac2 complex. His-DOCK2, Myc-ELMO1, and Myc-Rac2 alone (lanes 1, 3, and 5) or together with NA7-hf Nef (lanes 2, 4, and 6) were transiently expressed in HEK 293 cells as indicated. DOCK2 was precipitated from extracts (lanes 1 and 2) with Ni–NTA resin (lanes 3 and 4). Nef–DOCK2 was then precipitated with anti-FLAG affinity gel (lanes 5 and 6), and the epitope-tagged proteins were detected by immunoblotting and visualized by enhanced chemiluminescence. (B) Rac1 associates with HIV-1 Nef. Nef and associated proteins were isolated from extracts of HEK 293 cells transiently expressing DOCK2, ELMO1, and Rac1 either alone (lanes 1 and 4), with NA7-hf (lanes 2 and 5), or with a Nef variant containing a disrupted myristoylation signal (lanes 3 and 6). Nef and associated proteins were detected in anti-FLAG immunoprecipitates (lanes 1–3) and in extracts (lanes 4–6) by immunoblotting. (C) The interaction with DOCK2, ELMO1, and Rac2 is a conserved function of lentiviral Nef proteins. The ability of selected hf-tagged HIV-1 (lanes 1–3 and 5) and SIV mac239 (lane 4) Nef proteins to bind DOCK2, ELMO1, and Rac2 was determined as described in the legend to (B) above. The protein band in (C) indicated by the asterisk is the heavy chain of anti-FLAG mAb.

By analogy to previously described interactions among Rac, ELMO1, and CDM family proteins (Brugnera et al. 2002; Sanui et al. 2003b), our finding that DOCK2, ELMO1, and Rac2 copurified with HIV-1 Nef suggested that DOCK2 forms a ternary complex with ELMO1 and Rac2 and that Nef binds this complex. To investigate these possibilities, we attempted to reconstitute these interactions in human embryonic kidney 293 (HEK 293) cells. Although HEK 293 cells express endogeneous ELMO1, our initial studies revealed that the association of Nef with DOCK2 and Rac2 was significantly enhanced by ectopic expression of ELMO1 (data not shown). Thus, to determine whether ELMO1 and Rac2 copurify with DOCK2, DOCK2-containing complexes were purified from HEK 293 cells transiently expressing His-tagged DOCK2, Myc-tagged ELMO1, and Myc-tagged Rac2 via DOCK2 using Ni–NTA resin and eluted with imidazole. Immunoblotting revealed that ELMO1 and Rac2 copurified with DOCK2 (Figure 2A, lane 3), indicating that DOCK2 complexes with ELMO1 (DOCK2–ELMO1) and Rac2.

Subsequently, we asked whether ELMO1 and Rac2 are subunits of DOCK2–Nef complexes. DOCK2–Nef-containing complexes were isolated from HEK 293 cells transiently expressing His-tagged DOCK2, Myc-tagged ELMO1, Myc-tagged Rac2, and HA-FLAG epitope-tagged HIV-1 Nef (NA7-hf) via DOCK2 using Ni–NTA resin and eluted with imidazole (Figure 2A, lane 4). DOCK2–Nef complexes were then reisolated from this eluate via Nef by anti-FLAG immunoprecipitation. It is evident that ELMO1 and Rac2 also copurified with complexes containing both Nef and DOCK2 (Figure 2A, lane 6), thus supporting the possibility that HIV-1 Nef binds DOCK2–ELMO1 complexes that contain Rac2.

### Nef Targets Rac1 and Rac 2 Isoforms

Our mass spectroscopic analyses indicated that HIV-1 Nef associates with Rac2, but left open the possibility that it also targets Rac1. Therefore, we tested whether Nef can associate with Rac1 in the context of DOCK2 and ELMO1 using the same HEK 293 transient expression assay. Nef and its associated proteins were isolated from cell extracts by anti-FLAG immunoprecipitation and visualized by immunoblotting (Figure 2B). Nef formed readily detectable complexes incorporating Rac1 (Figure 2B, lane 2), while a mutant Nef protein unable to associate with membranes due to disruption of its N-terminal myristoylation signal (NA7_(G2_^_∇_^_HA)_), and therefore functionally defective, did not associate with DOCK2, ELMO1, or Rac1 (Figure 2B, lane 3). These results indicate that myristoylated Nef targets the Rac1 and Rac2 isoforms.

Nef proteins from well-characterized primate lentiviruses display considerable amino acid sequence variation. Therefore, we verified that Nef proteins from additional well-characterized laboratory HIV-1 strains (SF2 and NL4–3) bind DOCK2, ELMO1, and Rac2. We also tested a Nef protein from a strain of pathogenic SIV, mac239, that is important for rapid progression to AIDS in experimentally infected rhesus macaques (Kestler et al. 1991). Nef and its associated proteins were isolated from HEK 293 cell extracts by anti-FLAG immunoprecipitation and visualized by immunoblotting (Figure 2C). Functional Nef proteins from all lentiviral strains tested associated with DOCK2, ELMO1, and Rac2. This indicates that the ability to associate with Rac and its upstream regulators is a conserved function of primate lentiviral Nef.

### Nef Activates Rac in Resting T Cells

**Figure 3:**
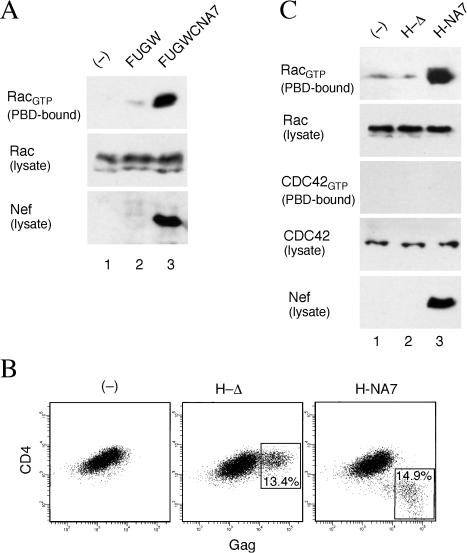
Nef Activates Rac in Resting CD4^+^ T Lymphocytes (A) HIV-1 Nef activates Rac in Jurkat T cells. Jurkat T cells (lane 1) were transduced with a control empty vector (FUGW; lane 2) or the same vector expressing HIV-1 NA7 Nef (FUGWCNA7; lane 3). Rac_GTP_ was precipitated from cell extracts with recombinant PAK1 PBD–GST. PBD–GST bound Rac_GTP_ (top), total Rac present in extracts (middle), and Nef (bottom) were detected by immunoblotting. (B) Flow cytometric analysis of Gag and CD4 expression in resting CD4^+^ T lymphocytes transduced with HIV-1 derived vectors in the presence of IL-7. Percentages of cells productively infected with *nef*-deleted H-Δ vector (boxed area in middle panel) or with HIV-1 NA7 *nef* containing H-NA7 vector (right panel) are shown. Results obtained with uninfected control CD4^+^ T cells cultured in the presence of IL-7 are also shown (left panel). (C) HIV-1 Nef specifically activates Rac in resting primary CD4^+^ T lymphocytes. Rac_GTP_ and CDC42_GTP_ were precipitated with PAK1 PBD–GST from extracts prepared from CD4^+^ T lymphocytes transduced with HIV-1 derived vectors, shown in (B), and analyzed as described in (A).

Since DOCK2, ELMO1, and Rac are major Nef-associated proteins in Jurkat T cells and since DOCK2 mediates Rac activation, we determined the effect of Nef on Rac activity in these cells. The active GTP-bound form of Rac (Rac_GTP_) binds the p21-binding domains (PBD) of PAKs directly (Burbelo et al. 1995). Hence, we used a PBD–GST fusion protein in pulldown assays to measure the fraction of activated Rac in vivo. Jurkat T cells were transduced with a lentiviral vector directing the expression of HIV-1 Nef (FUGWCNA7) or a control empty vector (FUGW). Extracts prepared from these cells were incubated with PBD–GST and the fraction of PBD-bound Rac was determined by immunoblotting (Figure 3A). Notably, the expression of Nef resulted in a readily detectable increase in the steady-state level of PBD-bound Rac (Figure 3A, lane 3), consistent with the possibility that the interaction of Nef with DOCK2–ELMO1 increases Rac activation.

In nontransformed T lymphocytes, Rac activation through DOCK2 is tied to chemotactic and antigenic stimuli. To assess whether Nef can uncouple these processes, we determined the effect of Nef on Rac activation in primary CD4^+^ T lymphocytes in the absence of stimulation with antigen and chemokines. While resting T cells are normally refractory to productive infection by lentiviruses and lentivirus-derived vectors, a sizable fraction becomes permissive for infection when cultured in the presence of cytokines such as IL-7 (Unutmaz et al. 1999). We used this procedure to infect primary resting CD4^+^ T lymphocytes with an HIV-1-derived vector expressing HIV-1 NA7 Nef (H-NA7) or a control *nef*-deleted vector (H-Δ). Since HIV-1 Env protein may activate DOCK2-controlled signaling pathways through binding to chemokine receptors such as CXCR4, *env*-defective, VSV-G-pseudotyped viruses were used in these experiments. We cultured 98% pure populations of CD4^+^ T cells isolated from the peripheral blood leukocytes of healthy donors for 5 d in the presence of IL-7 and then transduced them with Nef-expressing H-NA7 or control H-Δ virus. The purity of the infected populations and the efficiency of transduction were assessed 4 d later by flow cytometric analysis of CD4 expression on the cell surface and intracellular p24 Gag expression, respectively. As shown in Figure 3B, between 13% and 15% of CD4^+^ T cells were productively infected. Notably, the unusually low level of CD4 on the surface of cells infected with H-NA7 virus was due to robust downregulation of cell surface CD4 by NA7 Nef (Mariani and Skowronski 1993). Cell extracts were prepared from the infected populations, and PBD–GST pulldown assays were performed to determine the fraction of activated Rac. Strikingly, infection with H-NA7 resulted in a readily detectable increase in the steady state level of activated Rac (Figure 3C). Based on direct quantitations of chemiluminescent signals of total and PBD–GST bound Rac, we estimated that approximately 1.2% of the total Rac in extracts from cells transduced with H-NA7 was bound to PBD–GST as compared to 0.2% in extracts from cells transduced with H-Δ. The activation of Rac was specifically due to the expression of Nef and not other viral gene products, as infection with the otherwise isogenic H-Δ virus did not increase PBD–GST-reactive Rac. To address the specificity of Nef effect towards Rac, we then asked whether Nef affects activity of CDC42 GTPase, which also uses PAK as a downstream effector (Burbelo et al. 1995). Direct quantitations of chemiluminescent signals for total and PBD–GST-bound CDC42 revealed that less than 0.2% of the total CDC42 in extracts from H-NA7 and H-Δ transduced cells was PBD–GST bound. Therefore, we concluded that Nef primarily activates Rac and not CDC42 in CD4^+^ T lymphocytes in the absence of antigenic stimuli.

### Nef Activates Rac through DOCK2–ELMO1

**Figure 4:**
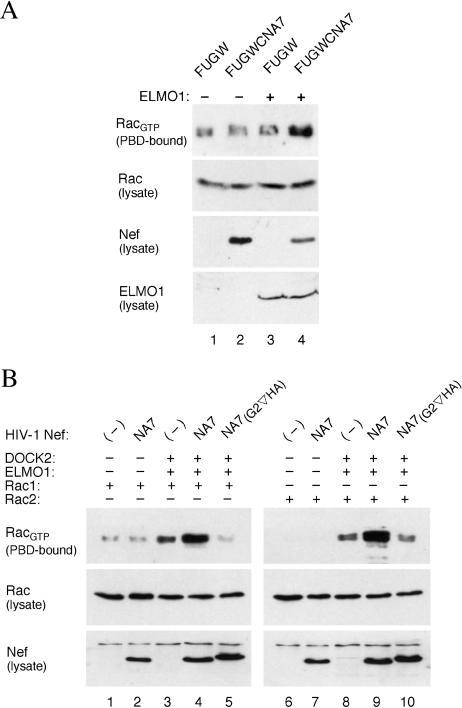
ELMO1 and DOCK2 Mediate Rac Activation by HIV-1 Nef (A) ELMO1 is required for Rac activation by Nef in NS1 cells. Rac_GTP_ and total Rac in the extracts prepared from ELMO1-deficient NS1 cells (lanes 1 and 2) and ELMO1-expressing NS1 cells (lanes 3 and 4) following transduction with a lentiviral vector expressing HIV-1 Nef (lanes 2 and 4) or a control empty vector (lanes 1 and 3) were visualized as described in the legend to Figure 3. (B) Nef activates Rac through DOCK2 and ELMO1 in HEK 293 cells. Rac_GTP_ and total Rac in the extracts prepared from HEK 293 cells coexpressing the indicated proteins were visualized as described above.

Next we asked whether Nef activates Rac through DOCK2–ELMO1. To determine whether ELMO1 is required for the effect of Nef, we measured Rac activation by Nef in NS1 lymphoma cells, which do not express the endogenous ELMO1 (Sanui et al. 2003b) and in NS1 cells in which ELMO1 expression was restored by retrovirus-mediated transfer of *ELMO1* cDNA (NS1_ELMO1_). NS1 and NS1_ELMO1_ cells were infected with a lentiviral vector expressing HIV-1 NA7 Nef (FUGWCNA7) or with a control empty vector (FUGW). Cell extracts were prepared from the infected populations, and PBD–GST pulldown assays were performed to determine the fraction of activated Rac. In agreement with a previous report (Sanui at al. 2003b), NS1 cells contain a small but readily detectable pool of activated Rac in spite of the lack of detectable ELMO1 expression, which is most likely generated by ELMO1-independent mechanism(s) (Figure 4A, lane 1). Notably, expression of Nef in the absence of ELMO1 and expression of ELMO1 in the absence of Nef did not increase the fraction of activated Rac (compare lane 2 to lane 1 and lane 3 to lane 1, respectively, in Figure 4A). In contrast, expression of Nef in the presence of ELMO1 induced a readily detectably increase in the pool of activated Rac in the NS1_ELMO1_ cells (Figure 4A, lane 4). These observations indicate that Nef activates Rac through an ELMO1-dependent mechanism.

Next, we studied Rac activation by Nef in HEK 293 cells, which do not express endogeneous DOCK2. Combinations of Nef, DOCK2, ELMO1, and either Rac1 or Rac2 were expressed in HEK 293 cells by transient transfection. The expression of Nef in the absence of DOCK2 had little effect on the activation of either Rac isoform (Figure 4B, lanes 2 and 7). Ectopic expression of DOCK2 and ELMO1 increased the fraction of activated Rac1 and Rac2 by approximately 3- to 4-fold (Figure 4B, lanes 3 and 8), which is in agreement with a previous report (Sanui et al. 2003b). Significantly, coexpression of myristoylated, but not unmyristoylated, Nef (Figure 4B, lanes 4 and 9 versus lanes 5 and 10) with DOCK2 and ELMO1 further enhanced the fraction of activated Rac isoforms by approximately 2-fold, and this effect of Nef was more pronounced for Rac2 (compare lane 9 with lane 4 in Figure 4B). Together, these data indicate that myristoylated Nef stimulates Rac activation through the DOCK2–ELMO1 complex.

### Nef Activates Rac through Association with DOCK2 and ELMO1

**Figure 5:**
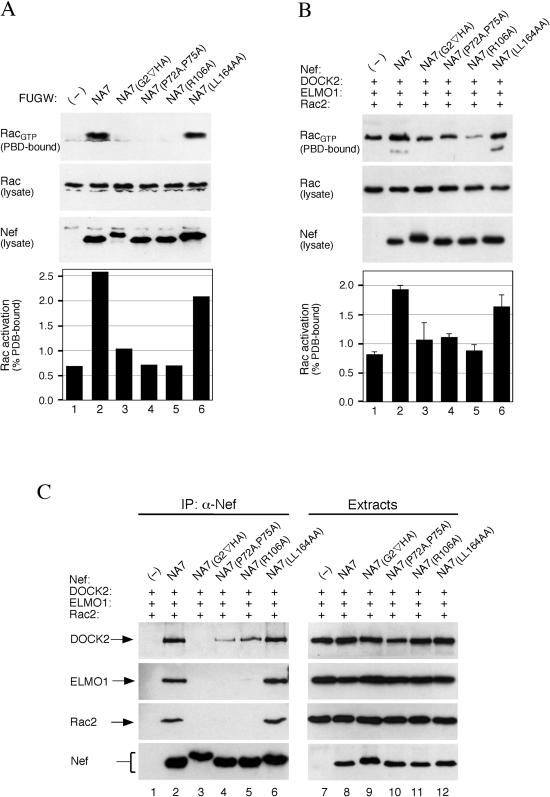
Nef Potentiates Rac Activation through Association with DOCK2–ELMO1 (A and B) Myristoylation signal, P72,P75, and R106 in Nef are required for Rac activation. Rac_GTP_ and total Rac in the extracts prepared from Jurkat T cells transduced with lentiviral vectors expressing no Nef (−) or the indicated Nef proteins (A) and HEK 293 cells transiently coexpressing the indicated Nef mutants together with DOCK2, ELMO1, and Rac2 (B) were visualized as described in the legend to Figure 3 and quantified by direct imaging of chemiluminescent signals. The fraction of total Rac present in the extracts that was PBD–GST bound is shown in the histograms. Data in the histogram shown in (B) are averages of three independent experiments and error bars represent two standard deviations. (C) Myristoylation signal, P72,P75, and R106 in Nef are required for association with DOCK2, ELMO1, and Rac2. The ability of selected Nef mutants to associate with DOCK2, ELMO1, and Rac2 was determined as described in Figure 2.

We identified mutations in Nef that disrupt Rac activation. Nef was reported to associate with an active form of PAK, and this interaction was suggested to be important for Nef effects on the cytoskeleton and T cell activation (Fackler et al. 1999; Arora et al. 2000; Wang et al. 2000). Since PAK is an immediate downstream effector of Rac, we asked whether the abilities of Nef to activate Rac through DOCK2–ELMO1 and to associate with activated PAK are correlated. Two different Nef mutations that were previously reported to disrupt its association with activated PAK (P72A,P75A and R106A) (Sawai et al. 1995; Renkema and Saksela 2000), yet unlike the myristoylation signal mutation (G2_∇_HA) did not significantly affect Nef functions in other assays, were tested for their effects on Rac activation in Jurkat T cells (Figure 5A) and in HEK 293 cells transiently expressing DOCK2, ELMO1, and Rac2 (Figure 5B). Both mutations abolished the ability of Nef to stimulate Rac activation (Figures 5A and 5B, lanes 4 and 5). As expected, the same was true for mutation of the myristoylation signal in Nef (Figures 5A and 5B, lane 3). In contrast, a mutation that specifically abrogates the interaction of Nef with clathrin adaptor proteins (LL164AA; Greenberg et al. 1998) had little disruptive effect on Rac activation (Figures 5A and 5B, lane 6).

Next we asked whether mutations in Nef that disrupted Rac2 activation affected the association with DOCK2, ELMO1, and Rac2 (Figure 5C). The LL164AA mutation, which did not significantly compromise the stimulation of Rac activation, did not affect the association of Nef with these proteins (Figure 5C, lane 6). In contrast, mutations that reduced Rac2 activation by Nef also diminished its association with DOCK2, ELMO1, and Rac2 (Figure 5C, lanes 3–5). Notably, the P72A,P75A and R106A mutations completely disrupted detectable association with ELMO1 and Rac2, but only weakened that with DOCK2, suggesting that Nef associates with both DOCK2 alone and DOCK2 complexed with ELMO1 and/or Rac2 and that the P72A,P75A and R106A mutations preferentially disrupt binding to the latter complex. Thus, robust stimulation of Rac2 activation by Nef requires its association with both DOCK2 and ELMO1.

### Functional Consequences of Nef Interactions with DOCK2–ELMO1 and Rac

The CD4^+^ T lymphocyte is a major target of infection by primate lentiviruses. Nef was reported to lower the threshold signal required for antigen-induced responses of T cells (Schrager and Marsh 1999; Wang et al. 2000), and this effect was proposed to be an important component to stimulation of viral replication by Nef in vivo (Alexander et al. 1994; Simmons et al. 2001). Since DOCK2 mediates Rac activation downstream of the TCR to modulate T cell responsiveness and downstream of chemokine receptors to mediate chemotactic responses (Fukui et al. 2001; Sanui et al. 2003a), we studied effects of Nef on these processes in T lymphocytes.

**Figure 6:**
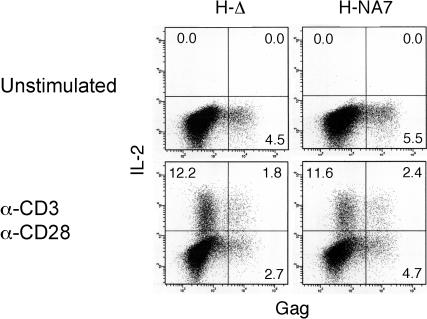
Effect of Nef on IL-2 Expression in HIV-1-Infected CD4^+^ T Lymphocytes Stimulated through CD3 and CD28 CD4^+^ T lymphocytes transduced with H-Δ and H-NA7 HIV-1-derived vectors were not stimulated (unstimulated) or stimulated with immobilized anti-CD3 and anti-CD28 mAbs (anti-CD3, anti-CD28) in the presence of Golgi-Stop for 5 h and stained for intracellular IL-2 and p24 Gag. Percentages of IL-2-positive and IL-2-negative cells in the Gag-negative and Gag-positive populations are shown.

Purified populations of primary resting CD4^+^ T cells were transduced with VSV-G-pseudotyped H-NA7 or *nef*-deleted H-Δ in the presence of IL-7. Cells were stimulated 4–6 d following transduction with plate-bound anti-CD3 and anti-CD28 antibodies, mixed at various ratios, for various amounts of time. Intracellular IL-2 and p24 Gag were visualized and quantified by flow cytometry to provide a measure of cellular activation of the infected cells in response to the stimulation. In the absence of anti-CD3/anti-CD28 stimulation, neither uninfected (Gag-negative) nor the productively infected (Gag-positive) cells, produced detectable amounts of IL-2 (Figure 6). In contrast, stimulation through CD3 and CD28 induced readily detectable accumulation of IL-2 in both H-Δ- and H-NA7-transduced populations. Notably, a larger fraction of productively infected CD4^+^ T lymphocytes typically proceeded to express IL-2 than uninfected cells. This phenomenon suggests that the permissive state for HIV-1 infection induced by IL-7 is associated with an increased responsiveness to activation via CD3 and CD28. However, no significant difference in the levels of IL-2 expression in cells infected with H-Δ compared to those infected with H-NA7 was detected across a wide range of stimulation conditions. These observations contrast with results from previous studies using T cell lymphoma and nontransformed CD4^+^ T lymphocytes expressing Nef alone (Schrager and Marsh 2000; Wang et al. 2001). This difference could, for example, reflect the modifying effect of other HIV-1 gene products that were not tested in the previous experiments. On the other hand, we cannot exclude the possibility that IL-7 treatment masks the effect of Nef. Since Nef deregulates DOCK2 function and DOCK2 is essential for proper signaling through the immunological synapse, further studies under a variety of conditions that modulate the formation and function of the immunological synapse may be required to reveal this effect of Nef in the context of HIV-1 infection.

**Figure 7:**
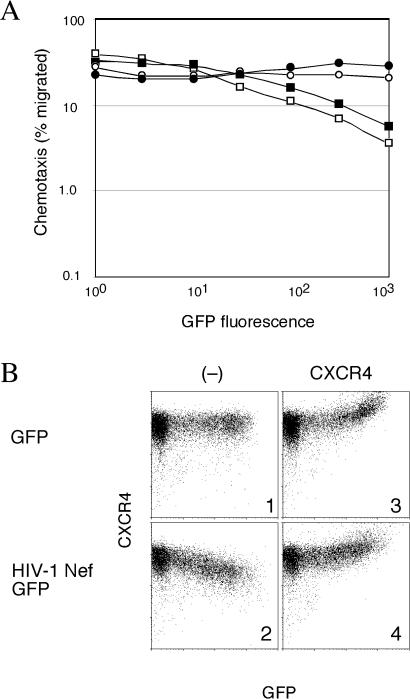
Nef Disrupts T Cell Migration to SDF-1 (A) Migration of cell populations shown in (B) expressing GFP (open circle), ectopic CXCR4, and GFP (filled circle), HIV-1 NA7 Nef and GFP (open box), HIV-1 NA7 Nef, ectopic CXCR4 and GFP (filled box) to SDF-1 was measured in transwell assays. (B) Transient expression of ectopic CXCR4 restores CXCR4 levels on the surface of Nef-expressing cells. Flow cytometric analysis of Jurkat T cells transiently expressing GFP (panel 1) or HIV-1 NA7 Nef and GFP (panel 2) and together with ectopic CXCR4 (panels 3 and 4, respectively).

DOCK2 regulates the activation of Rac proteins during lymphocyte migration in response to chemokine gradients (Fukui et al. 2001). Therefore, we also asked whether Nef affects lymphocyte chemotaxis. Jurkat T cells, which constitutively express CXCR4, a major coreceptor for T-cell tropic HIV and a receptor for stromal-derived factor 1 (SDF-1) (Deng et al. 1996; Feng et al. 1996), were transiently transfected with a control plasmid expressing enhanced green fluorescent protein (GFP) alone or with a plasmid that coexpresses Nef and a GFP marker protein from the same bicistronic transcription unit. We then measured the chemotaxis of transfected populations to SDF-1 using a transwell migration assay. The relative frequency of control and Nef-expressing cells in the migrated populations was determined by flow cytometric measurement of GFP expression. Approximately 30% of control cells migrated regardless of the level of GFP expression, indicating that the chemotaxis in this assay was robust (Figure 7A). In contrast, the chemotaxis of cells coexpressing Nef and GFP was inhibited in a dose-dependent manner.

Control experiments revealed that Nef caused a modest decrease in cell surface expression of CXCR4 (compare panels 1 and 2 in Figure 7B). This observation raised the possibility that Nef-expressing cells were unresponsive to SDF-1 due to abnormally low levels of CXCR4 at the cell surface rather than due to deregulation of the DOCK2–ELMO1 complex. To address this possibility, we restored CXCR4 on the surface of Nef-expressing cells to levels equal to and even higher than those seen in control cells by transiently expressing ectopic CXCR4 receptor and then performed migration assays using these cell populations (Figure 7B, panels 3 and 4). Significantly, the migration of cells with restored CXCR4 levels was still inhibited by Nef expression (Figure 7A). We concluded that HIV-1 Nef blocks lymphocyte migration to SDF-1 principally by interfering with CXCR4-controlled signaling cascades rather than by downregulating CXCR4 from the cell surface.

**Figure 8:**
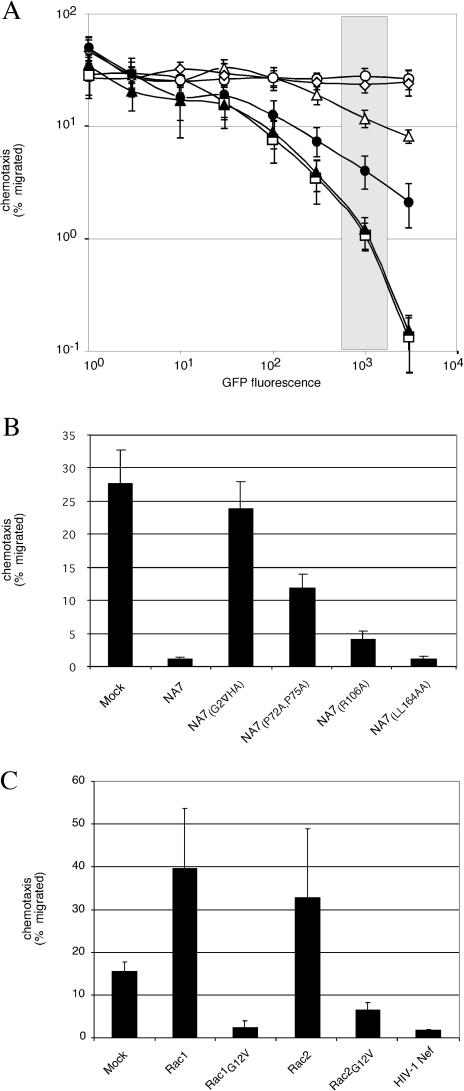
Nef Disrupts Chemotaxis by Activating Rac through DOCK2–ELMO1 (A and B) Jurkat T cells expressing wild-type or mutant HIV-1 Nef proteins and GFP reporter were used in transwell chemotaxis assays with SDF-1. Percentage of migrated cells expressing GFP alone (open circle), or together with HIV-1 NA7 (open square), NA7_(G2__^∇^__HA)_ (open diamond), NA7_(P72A,P75A)_ (open triangle), NA7_(R106A) _(filled circle), and NA7_(LL164AA)_ (filled triangle) is shown as a function of GFP fluorescence intensity in (A) and in (B) for the single GFP fluorescence intensity interval indicated by the shaded rectangle in (A). (C) Constitutively active Rac GTPases disrupt lymphocyte migration to SDF-1. Migration of Jurkat T cells transiently expressing wild-type (Rac1, Rac2), constitutively active (Rac1_G12V_, Rac2_G12V_), or as a control HIV-1 Nef were also measured. Data shown are averages of three independent experiments and error bars represent two standard deviations.

We then asked whether inhibition of Jurkat T cell migration by Nef correlated with its ability to potentiate Rac activation by DOCK2 and ELMO1. As expected, we found that disruption of the myristoylation signal in Nef (NA7_(G2__^∇^__HA)_) abolished the inhibition of migration (Figure 8A and 8B). Furthermore, the P72A,P75A, and R106A mutations diminished the ability of Nef to block migration, albeit to different extents. In contrast, the LL164AA mutation, which had little disruptive effect on enhancement of Rac activation, was fully functional in this assay. These observations suggested that deregulated activation of Rac GTPases is instrumental for the defective migration of Nef-expressing cells. To explore this possibility further, we ectopically expressed constitutively active Rac1 and Rac2 (Rac1_G12V_, Rac2_G12V_) and, as controls, wild-type Rac1 and Rac2 in Jurkat T cells and measured their migration to SDF-1 (Figure 8C). Expression of wild-type Rac GTPases stimulated chemotaxis approximately 2- to 3-fold. In contrast, expression of the constitutively active forms of each Rac GTPase severely suppressed lymphocyte migration to SDF-1. Thus, deregulated Rac activation inhibits directional cell movement, most likely by disrupting spatially organized rearrangements of the cytoskeleton that are induced by chemokine gradients. These results further support a model in which Nef disrupts migration to SDF-1 by activating Rac through the DOCK2–ELMO1 module and thus uncoupling Rac activation from chemokine receptor signaling.

**Figure 9:**
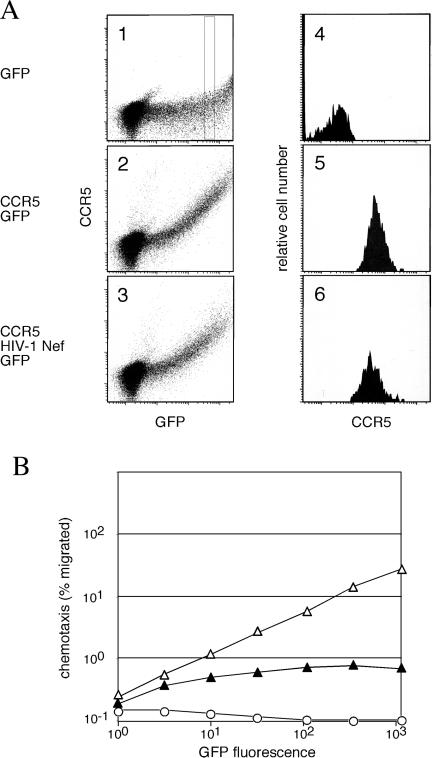
HIV-1 Nef Disrupts CCR5-Mediated Migration (A) HIV-1 Nef does not downregulate CCR5. Flow cytometric analysis of CCR5 and GFP in Jurkat T cells transiently expressing GFP alone (panel 1) or CCR5 and GFP in the absence (panel 2) and presence (panel 3) of HIV-1 Nef. Histograms of CCR5 expression for cell populations within a single GFP fluorescence intensity interval indicated by the rectangle in panel 1 are shown in panels 4 to 6, respectively. (B) Percentage of cells migrated to MIP-1β and expressing GFP alone (open circle), GFP and CCR5 (open triangle), or GFP, CCR5 and HIV-1 Nef (filled triangle) is shown as a function of GFP fluorescence intensity.

The above observations predicted that Nef likely causes a general migration defect. Therefore, we also studied the effect of Nef on Jurkat T cell migration to the MIP-1β chemokine. Since the MIP-1β receptor (CCR5) is not constitutively expressed in Jurkat T cells, we transiently expressed CCR5 and GFP marker from a bicistronic vector either alone or together with HIV-1 NA7 Nef. Using this vector, CCR5 expression levels were positively correlated with GFP marker protein expression (Figure 9A, panels 2 and 3). Notably, flow cytometric analysis revealed that CCR5 cell-surface expression was not downregulated by Nef (compare panels 5 and 6 in Figure 9A). We then measured the ability of these cells to migrate to MIP-1β. Migration of cells coexpressing CCR5 and HIV-1 Nef was impaired compared to control cells expressing CCR5 at comparable levels (Figure 9B). These data confirm that Nef induces a general defect in lymphocyte migration by targeting DOCK2–ELMO1 and Rac.

## Discussion

Nef is a multifunctional adaptor protein that modulates signal transduction and protein-sorting machineries. We purified to near homogeneity an abundant Nef-associated protein complex from T cells and identified by mass spectroscopy its major subunits as DOCK2 and ELMO1, a bipartite Rac activator (Sanui et al. 2003b). Notably, the extensive large-scale biochemical purification and sensitive proteomic analyses described in this report did not detect several cellular proteins previously reported to associate with HIV-1 Nef and mediate the effects of Nef in the cell (data not shown). This includes cellular proteins such as Vav (Fackler et al. 1999), PAKs (Sawai et al. 1995), inositol triphosphate receptor (Manninen and Saksela 2002), Lck protein-tyrosine kinase (Baur et al. 1997), clathrin adaptors (Greenberg et al. 1997; Le Gall et al. 1998; Piguet et al. 1998), and others.

Why were proteins previously reported to associate with Nef not detected in our studies? One possible explanation is that previously reported associations are unstable under the biochemical purification conditions used in our studies. This likely explains the apparent absence of clathrin adaptors in Nef preparations purified in our studies, since we know that Nef binds AP-2/AP-1 clathrin adaptors only weakly in the salt and pH conditions used here (data not shown). It is also possible that in some cases, epitope tags may be buried and therefore inaccessible to the monoclonal antibodies (mAbs) used for immunoaffinity purification. Moreover, some of the previously reported associations, especially those with protein kinases, such as PAKs, were best detected by an ultrasensitive in vitro kinase assay (Sawai et al. 1995) and, as our data show, are likely of exceedingly low stoichiometry. (Of note, we did detect the presence of p62 phosphoprotein [PAK] by in vitro kinase assays of anti-Nef purifications [data not shown]). Finally, some of the described associations, such as that with thioesterase, are known to occur only with selected Nef variants (Cohen et al. 2000), while those we report here occur with all Nef variants tested. Nonetheless, the specific isolation of the Nef–DOCK2–ELMO1–Rac complex reported here provides strong biochemical evidence to reinforce predictions from previous genetic studies that Nef functions through multiple independent interactions with different sets of downstream effector proteins.

Our observations strongly argue that DOCK2–ELMO1 is the major upstream regulator used by Nef to activate Rac in T cells and that through this interaction Nef can activate Rac in CD4^+^ T lymphocytes even in the absence of stimulation with antigen or chemokines. These data indicate that Nef targets a critical switch, DOCK2–ELMO1, that regulates Rac GTPases downstream of chemokine receptors and the TCR and uses it to modulate the downstream processes they control. Thus, the interaction of Nef with DOCK2 and ELMO1 provides an important mechanism by which Nef may impact pathogenesis by primate lentiviruses.

Our data strongly suggest that DOCK2–ELMO1 is the major activator of Rac targeted by Nef in T lymphocytes. This model is supported by the observations that Nef physically associates with a complex that contains DOCK2–ELMO1 and Rac, that specific mutations in Nef simultaneously disrupt its ability to bind this complex and to activate Rac, and that Nef fails to activate Rac in the absence of ELMO1. Although previous reports implicated Vav, a Rac1 guanine nucleotide exchange factor (GEF), as the critical downstream effector that Nef binds directly to activate PAK (Fackler et al. 1999), our data do not support this possibility. Notably, we have been unable to detect the presence of Vav in anti-Nef immune complexes by both proteomic analyses and immunoblotting, indicating that these interactions are of low abundance relative to those with DOCK2 and ELMO1 and are therefore unlikely to mediate the bulk of Nef's impact on the Rac pathway. Significantly, ELMO1 is ubiquitously expressed (Gumienny et al. 2001) and can associate with Nef in nonlymphoid cells in the absence of DOCK2 (data not shown). Thus, we postulate a general mechanism in which ELMO1, possibly in complex with another CDM family protein, mediates Rac activation and PAK recruitment by Nef that is observed in nonlymphoid cells (Sawai et al. 1995; Fackler et al. 1999; Arora et al. 2000).

Our observation that the expression of Nef from the integrated HIV-1 provirus in primary CD4^+^ T cells did not alter IL-2 production in standard activation protocols was unexpected, because previous genetic evidence linked Nef binding to DOCK2–ELMO1 and the ensuing activation of Rac GTPases to the the ability of Nef to facilitate T cell activation. Specifically, the same mutations that we observed to disrupt Rac activation through the DOCK2–ELMO1 complex were previously reported to disrupt the stimulatory effect of Nef on aspects of T cell activation (Sawai et al. 1995; Simmons et al. 2001), and the observed effects were in some cases dramatic (Wang et al. 2000). Moreover, Rac activation by DOCK2 facilitates T cell responsiveness to antigen, as disrupted Rac activation in *DOCK2*(−/−) and *Rac2*(−/−) mice is associated with defective immunological synapse formation and depressed antigen-specific responses (Yu et al. 2001; Sanui et al. 2003a). Since Nef deregulates DOCK2 function and DOCK2 is essential for proper signaling through the immunological synapse, further studies under a variety of conditions that modulate the formation and function of the immunological synapse may be required to reveal the effect of Nef in the context of HIV-1 infection.

Interestingly, the chemokine receptor system plays crucial roles in infection by primate lentiviruses. Previous studies revealed its essential role for virus entry into target cells (Deng et al. 1996; Feng et al. 1996). Primate lentiviruses also exploit this system to recruit uninfected target cells to sites of viral replication (Weissman et al. 1997; Swingler et al. 1999). Our observations reveal an additional level of complexity in primate lentivirus–chemokine receptor system interactions. Inhibiting chemotaxis of the infected T cells likely disrupts the generation of the immune response. During generation of the immune response, T cells are initially activated in the paracortex of lymph nodes and then migrate to the edges of follicles, where they interact with antigen receptor-activated B cells (Garside et al. 1998). This physical interaction is required to drive B cells towards antibody production, isotype switching, and the affinity maturation of the antibody response. Hence, the development and maturation of the immune response require the ordered migration of activated T cells to specific sites within lymphoid tissue (Delon et al. 2002). Notably, recent in vivo evidence documents Nef-dependent alterations in the distribution of SIV mac239-infected CD4^+^ T lymphocytes in the lymph nodes of experimentally infected rhesus macaques (Sugimoto et al. 2003). In lymph nodes of monkeys infected with *nef-*deleted SIV, most infected T cells were located in the B cell-rich follicles and in the border region between the paracortex and the follicles. In contrast, in monkeys infected with SIV harboring a functional *nef* gene, most productively infected T cells remained in the T cell-rich paracortex and were only infrequently present in proximity to B cell follicles. This evidence shows that Nef disrupts the ordered migration patterns of infected CD4^+^ T lymphocytes in vivo and reinforces the possibility that this disruption impairs the generation and maturation of the immune response to antigens. Since a large fraction of HIV-1-infected CD4^+^ T cells is specific for HIV-1 antigens (Douek et al. 2002), this effect of Nef provides another mechanism to suppress the antiviral immune response. Taken together, the functional interaction of Nef with DOCK2, ELMO1, and Rac enables Nef to modulate multiple aspects of T cell function.

##  Materials and Methods 

### 

#### Construction of expression plasmids

Sequences encoding variant and mutant Nef proteins tagged at their C-termini with a peptide (hf) containing the HA and FLAG epitopes (DTYRYIYANATYPYDVPDYAGDYKDDDDK) were subcloned into pBABE-*puro* and pCG expression plasmids (Morgenstern and Land 1990; Greenberg et al. 1998). In NA7_(G2__∇__HA)_ Nef, the myristoylation signal was disrupted by insertion of the HA epitope (ANATYPYDVPDYAG) at glycine G2. The full-length human *ELMO1* cDNA (clone IMAGE:4521393; ResGen, Carlsbad, California, United States), *DOCK2* cDNA (KIAA0209, clone ha04649; Kazusa DNA Research Institute, Chiba, Japan), and cDNAs encoding wild-type and mutant forms of Rac1 and Rac2 (kindly provided by Linda Van Aelst, Cold Spring Harbor Laboratory, Cold Spring Harbor, New York, United States) were subcloned into pCG plasmids containing N-terminal c-Myc (EQKLISEEDL) or polyhistidine (HHHHHHH) epitope tags, using standard techniques. pBABE *ELMO1* contains an N-terminal c-Myc epitope-tagged *ELMO1* cDNA subcloned into the pBABE-*neo* vector. H-NA7 was constructed by substituting the HIV-1 NL4–3 *nef* coding sequence with that of HIV-1 NA7 *nef* (Mariani and Skowronski 1993) in pNL4–3 carrying a frameshift mutation at the KpnI site at position 6463 in *env*, kindly provided by Klaus Strebel (National Institute of Allergy and Infectious Diseases, National Institutes of Health, Bethesda, Maryland, United States). H-Δ is based on H-NA7 with a deletion that removes residues 1–34 of Nef and prevents expression of Nef protein. For chemotaxis assays, cDNAs encoding HIV-1 NA7 Nef, human CCR5 (kindly provided by Frank Kirchhoff, Universitätsklinikum, Ulm, Germany), and Rac proteins were subcloned into the pCGCG bicistronic vector that directs the expression of GFP from an internal ribosomal entry site element (Lock et al. 1999).

#### Generation of stable cell lines

pBABE plasmids were introduced into the Phoenix amphotropic packaging cell line, kindly provided by G. P. Nolan (Stanford University Medical Center, Palo Alto, California, United States), by calcium phosphate coprecipitation, and viral supernatants were used to infect a Jurkat T cell subline (Greenberg et al. 1998), provided by Dan R. Littman (New York University School of Medicine, New York, New York, United States), or NS1 cells. Transduced Jurkat cells were selected and subsequently maintained in the presence of puromycin (0.4 μg/ml) (Sigma, St. Louis, Missouri, United States). Transduced NS1 cells were selected and maintained in the presence of G418 (1.0 mg/ml) (Invitrogen, Carlsbad, California, United States).

#### Immunoaffinity purification of epitope-tagged Nef and associated proteins

Unless stated otherwise, all reactions were performed at 4°C. Approximately 1.8 × 10^10^ Jurkat T cells stably expressing NA7-hf (or control cells) were lysed for 1 h in 200 ml of LB buffer (150 mM NaCl, 50 mM Tris–HCl [pH 7.5], 1% Triton X-100, 10% glycerol) (Complete Protease Inhibitors, Roche, Basel, Switzerland). Extracts were precleared with protein G–agarose (Roche) for 1 h and incubated with 12CA5 mAb crosslinked to protein G–agarose beads for 4 h. The immunoprecipitate was washed extensively with LB, and bound proteins were eluted by competition with HA peptide (0.2 mg/ml) (ANATYPYDVPDYAG; Invitrogen) in LB for 45 min at 30°C. The eluate was incubated with anti-FLAG M2 affinity gel beads (Sigma) overnight, and the immunoprecipitate was washed extensively with LB and then LB modified to contain 0.1% Triton X-100 (FEB buffer). Proteins were eluted with FEB containing FLAG peptide (0.2 mg/ml) (Sigma) for 45 min at 30°C. The eluate was concentrated on Microcon centrifugal filter devices (Millipore, Billerica, Massachusetts, United States) with a cutoff of 8 kDa.

#### Protein identification by mass spectrometry

Nef and associated proteins were separated by 8%–17% SDS-PAGE and visualized using SYPRO stain (Molecular Probes, Eugene, Oregon, United States). Visible bands were excised and processed for identification by mass spectrometry. The samples were analyzed by LC-MS/MS as described previously (Hu et al. 2002). Spectra resulting from LC/MS/MS were analyzed with the SONARS software package (ProteoMetrics LLC, New York, New York, United States).

#### Lentiviral vectors and infections

FUGWCNA7 contains the wild-type HIV-1 NA7 *nef* coding sequence under control of the CMV promoter, subcloned downstream of the Woodchuck-responsive element in the FUGW lentiviral vector (Lois et al. 2002). FUGWC vectors containing amino acid substitutions in Nef have a similar structure. Supernatants containing infectious particles were produced by calcium phosphate cotransfection of HEK 293 cells, as described previously (Lois et al. 2002). For biochemical analyses of Rac activation, approximately 10^7^ Jurkat T cells or NS1 cells were infected with supernatants containing approximately 10^7^ infectious units of FUGW or FUGWC vectors encoding wild-type or mutant HIV-1 NA7 Nef proteins, in the absence of polycationic agents. Cell extracts were prepared 3–4 d following infection and used for PBD–GST pulldown assays. PBMCs were purified from buffy coats of healthy donors (New York Blood Bank, Hicksville, New York, United States) by density gradient separation on Lymphocyte Separation Medium (ICN Biomedicals, Inc., Irvine, California, United States), and CD4^+^ T lymphocytes were isolated using CD4^+^ T Cell Enrichment Columns (R&D Systems, Minneapolis, Minnesota, United States). Replication incompetent HIV-1 particles pseudotyped with VSV-G were produced by calcium phosphate transfection of HEK 293 cells and used to infect >98% pure populations of CD4^+^ T lymphocytes that were cultured in the presence of IL-7 (Unutmaz et al. 1999). Cell-surface CD4 in the infected populations was revealed with FITC-conjugated SK3 mAb (Becton Dickinson, San Jose, California, United States). Cells stained for CD4 were permeabilized using Cytofix/Cytoperm Kit (BD PharMingen, San Jose, California, United States) and p24 Gag expression was revealed with PE-conjugated KC57-RD1 mAb (Beckman Coulter, Inc., San Diego, California, United States) as described elsewhere (Mascola et al. 2002). CD4 and Gag expression were quantitated simultaneously using an LSR-II flow cytometer (Becton Dickinson).

#### Cell stimulations and IL-2/Gag assays

Anti-CD3 mAb and anti-CD28 mAb (MAB100 and MAB342; R&D Systems), alone or in combinations, were immobilized on 12-well microtiter plates (351143; Becton Dickinson) in PBS overnight at 4°C. Wells were washed three times with PBS, and 5 × 10^5^ CD4^+^ T lymphocytes were added 4–5 d after they were transduced with H-Δ or H-NA7 vectors in the presence of IL-7. In some experiments, transduced cells were cultured for additional 48 h in the absence of IL-7 before stimulations. Stimulations were performed for 4 h to 16 h in the presence and absence of Golgi-Stop (Becton Dickinson). Cells were recovered from wells by vigorous pipetting, fixed, and permeabilized using Cytofix/Cytoperm Kit (BD Bioscience PharMingen). Intracellular IL-2 and p24 Gag were revealed simultaneously with PE-conjugated rat anti-human IL-2 mAb, (559334; B&D Biosciences PharMingen) and FITC-conjugated KC57-RD1 mAb (Beckman Coulter, Inc.), respectively.

#### Transient transfections of HEK 293 cells, immunoprecipitations, and immunoblotting

HEK 293 cells were transfected by calcium phosphate coprecipitation with 20 μg of pCG plasmids expressing epitope-tagged DOCK2, ELMO1, Rac2 or Rac1, and/or tagged Nef proteins and a control empty vector. Cells were lysed 48 h posttransfection in LB buffer. To isolate Nef and associated proteins, extracts were incubated overnight with anti-FLAG M2 Affinity Gel (Sigma), the immunoprecipitates were washed four times with LB buffer, once with LB containing 0.5 M LiCl, and proteins were eluted with FLAG peptide as described above. To isolate DOCK2 and associated proteins, extracts were incubated with Ni–NTA agarose (Qiagen, Valencia, California, United States) and proteins were eluted from the precipitate with 250 mM imidazole. DOCK2–Nef complexes were isolated from imidazole eluates with anti-FLAG M2 affinity gel as described above. Eluted proteins proteins were separated by 16% SDS-PAGE, electroblotted onto PVDF membrane (Millipore), and immunoblotted with the following antibodies: anti-c-Myc mAb (1:100; Oncogene, Tarrytown, New York, United States), anti-FLAG M2 mAb (1:5000; Sigma), 12CA5 mAb (1:5000), or rabbit serum raised to a DOCK2-specific peptide (GDKKTLTRKKVNQFFKTM). Immune complexes were revealed with HRP-conjugated antibodies specific for the Fc fragment of mouse or rabbit immunoglobulin G. (1:5,000; Jackson ImmunoResearch Laboratories, Inc., West Grove, Pennsylvania, United States) and ECL (Amersham, Little Chalfort, United Kingdom).

#### Rac and CDC42 activity assays

Cells were lysed in LB modified to contain 500 mM NaCl, 0.5% sodium deoxycholate, 0.1% SDS, and 10 mM MgCl_2_ (RB buffer). Extracts were incubated for 2 h with 40 μg of recombinant PAK1 PBD–GST (kindly provided by Linda Van Aelst) bound to glutathione–agarose beads (8 mg/ml) (Roche), and beads were washed extensively with RB buffer. Bound proteins and aliquots of extracts were separated by 16% SDS-PAGE. Rac was immunoblotted with anti-c-Myc epitope mAb (1:100; Oncogene), or with anti-Rac mAb (1:1000; BD Transduction Laboratories), CDC42 was detected with sc-87 rabbit antibody (1:100; Santa Cruz Biotechnology, Santa Cruz, California, United States), and Nef was detected with rabbit serum raised against HIV-1 HxB3 Nef (1:300; Mariani and Skowronski 1993). Immune complexes were visualized by chemiluminescence using Lumi-Lite Plus (Roche). Chemiluminescent signals were imaged and quantitated using the FluorChem Imaging System and software (Alpha Innotech, Cannock, United Kingdom).

#### Chemotaxis assays

Jurkat T cells were transfected by electroporation with plasmids coexpressing Nef and GFP marker protein from a single bicistronic transcription unit (pCGCG NA7; Lock et al. 1999), except that in some experiments were also cotransfected with pCG CXCR4, expressing human CXCR4 receptor. Transfected populations were used 24–48 h later in transwell chemotaxis assays in the presence of 10 ng/ml of SDF-1 (R&D Research). For CCR5-dependent migration, Jurkat T cells were transfected with pCGCG CCR5, expressing human CCR5 and GFP, alone or in the presence pCG NA7, and migration to MIP-1β (10 ng/ml) was measured. Cells were allowed to migrate for 2 h, and the relative frequency of GFP-positive cells in the initial and migrated populations was determined by flow cytometry using an LSR-II flow cytometer (Becton Dickinson).

## Supporting Information

### Accession Numbers

The LocusLink (http://www.ncbi.nlm.nih.gov/LocusLink/) accession numbers of the genetic loci discussed in this paper are *DOCK2* (LocusLink ID 1794), *ELMO1* (LocusLink ID 9844), *Rac1* (LocusLink ID 5879), and *Rac2* (LocusLink ID 5880).

## Abbreviations

CCR5: chemokine (CC motif) receptor 5
CDM: CED-5/DOCK180/Myoblast City
CXCR4: chemokine (CXC motif) receptor 4
DOCK: dedicator of cytokinesis
ELMO: engulfment and cell motility
GEF: guanine nucleotide exchange factor
GFP: green fluorescent protein
GST: glutathione S-transferase
HEK: human embryonic kidney
LC/MS/MS: liquid chromatography tandem mass spectroscopy
mAb: monoclonal antibody
PAK: p21-activated kinase
PBD: p21-binding domain
SDF-1: stromal-derived factor-1
TCR: T cell antigen receptor
